# Inter-kingdom effect on epithelial cells of the N-Acyl homoserine lactone 3-oxo-C12:2, a major quorum-sensing molecule from gut microbiota

**DOI:** 10.1371/journal.pone.0202587

**Published:** 2018-08-29

**Authors:** Cécilia Landman, Jean-Pierre Grill, Jean-Maurice Mallet, Philippe Marteau, Lydie Humbert, Eric Le Balc’h, Marie-Anne Maubert, Kevin Perez, Wahiba Chaara, Loic Brot, Laurent Beaugerie, Harry Sokol, Sophie Thenet, Dominique Rainteau, Philippe Seksik, Elodie Quévrain, on behalf of the Saint Antoine IBD Network

**Affiliations:** 1 Sorbonne Université, École normale supérieure, PSL University, CNRS, INSERM, APHP, Hôpital Saint-Antoine, Laboratoire des biomolécules, LBM, Paris, France; 2 Sorbonne Universités, INSERM, Immunology-Immunopathology-Immunotherapy (i3), Paris, France; 3 Sorbonne Universités, Centre de Recherche des Cordeliers, PSL University, EPHE, Paris, France; Consejo Superior de Investigaciones Cientificas, SPAIN

## Abstract

**Background and aims:**

N-acyl homoserine lactones (AHLs), which are autoinducer quorum-sensing molecules involved in the bacterial communication network, also interact with eukaryotic cells. Searching for these molecules in the context of inflammatory bowel disease (IBD) is appealing. The aims of our study were to look for AHL molecules in faecal samples from healthy subjects (HS) and IBD patients to correlate AHL profiles with the microbiome and investigate the effect of AHLs of interest on epithelial cells.

**Methods:**

Using mass spectrometry, we characterised AHL profiles in faecal samples from HS (*n* = 26) and IBD patients in remission (*n* = 24) and in flare (*n* = 25) and correlated the presence of AHLs of interest with gut microbiota composition obtained by real-time qPCR and 16S sequencing. We synthesised AHLs of interest to test the inflammatory response after IL1β stimulation and paracellular permeability on Caco-2 cells.

**Results:**

We observed 14 different AHLs, among which one was prominent. This AHL corresponded to 3-oxo-C12:2 and was found significantly less frequently in IBD patients in flare (16%) and in remission (37.5%) versus HS (65.4%) (*p* = 0.001). The presence of 3-oxo-C12:2 was associated with significantly higher counts of Firmicutes, especially *Faecalbacterium prausnitzii*, and lower counts of *Escherichia coli*. In vitro, 3-oxo-C12:2 exerted an anti-inflammatory effect on Caco-2 cells. Interestingly, although 3-oxo-C12, the well-known AHL from *Pseudomonas aeruginosa*, increased paracellular permeability, 3-oxo-C12:2 did not.

**Conclusions:**

We identified AHLs in the human gut microbiota and discovered a new and prominent AHL, 3-oxo-C12:2, which correlates with normobiosis and exerts a protective effect on gut epithelial cells.

## Introduction

Recent data highlighted the role of the host-microbiota interactions in the pathophysiology of inflammatory bowel disease (IBD)[1,2]. Convergent studies have shown that IBD patients exhibit dysbiosis (i.e., a disruption of the gut ecosystem equilibrium), characterised by restricted biodiversity, temporal instability and a quantitative decrease of bacteria belonging to the Firmicutes phylum, especially *Faecalibacterium prausnitzii*[3–6]. Beyond bacterial signatures for IBD, it is worthwhile to explore the functional consequences of IBD-associated dysbiosis on gut inflammation. Among the many molecules from the gut microbiota that share activities or functions in the gut ecosystem, those involved in the dialog with the host are of special interest[7–9].

Bacterial cell-cell communication, also known as quorum sensing (QS), relies on the ability of bacteria to monitor and respond to microbial density via diffusible signal molecules called autoinducers. This process allows bacterial populations to coordinate gene expression and behaviour, such as bioluminescence, secretion of virulence factors or biofilm formation. The best-known type of QS is mediated by small amphiphilic molecules, called N-Acyl homoserine lactones (AHLs). Mostly found in Gram-negative bacteria, they are biosynthesised by a LuxI-type synthase[10,11]. Then, AHLs diffuse freely in and out of the cell and in the extracellular medium. Once the bacterial density reaches the quorum, a threshold concentration of AHLs is also reached, and they are recognised by their cognate intracellular LuxR-type receptors, triggering a signal transduction cascade that results in population-wide changes in gene expression[12,13]. Pathogens can use AHLs as a virulence factor per se to modulate host immune responses, which has been well described with 3-oxo-C12-AHL from *Pseudomonas aeruginosa*[14].First considered to be a bacterial communication network only, QS driven by AHLs also proved to be involved in an inter-kingdom communication, i.e., host-bacteria interactions[15]. Importantly, paraoxonases (PON) are enzymes expressed in eukaryotic cells that are able to exert a ‘quorum quenching’ activity while hydrolysing AHLs’ ring moiety[16]. Interestingly, recent studies explored PON-1 activity in IBD patients[17,18].

QS driven by AHLs has been described in many bacterial ecosystems. However, they had never been studied in the human intestinal microbiota[19]. We hypothesised that QS could be involved in ecological changes in IBD, such as dysbiosis, and could also impact gut inflammation. The aim of our study was first to describe faecal AHLs in IBD patients compared to healthy subjects (HS) in parallel with gut microbiota composition. We developed original extraction and detection methods, and then we investigated the effect on epithelial cells of 3-oxo-C12:2, a novel and prominent AHL identified from the human gut ecosystem.

## Materials and methods

### Patients and samples

We collected faecal samples from IBD patients and healthy volunteers who provided informed consent. None of them had a history of bowel resection and none had taken antibiotics within three months before the sampling. Faecal samples were frozen within one hour and stored at -80°C until analysis. Demographic data, disease characteristics, disease activity and biological features at the time of sampling were collected.

### AHL detection

#### Sample pre-treatment and protocol for AHL extraction

Each faecal sample was lyophilised for 72 hours, and AHLs were extracted from one gram of lyophilised faeces according to the following steps: First, solid-liquid extraction was conducted using a Soxhlet apparatus with 100 mL of high performance liquid chromatography (HPLC)-grade ethyl acetate (Carlo Erba). Two μL of the internal standard N-hexanoyl-L-homoserine lactone-d3 (C6d3-HSL, Cayman Chemical) at 20 mM was added, and the process was left to run for 4 hours. Second, the extraction product was evaporated using a rotatory evaporator and resuspended in 5 mL of HPLC-grade acetonitrile (Carlo Erba). Then, 45 mL of water was added (90% water/10% acetonitrile). Third, solid phase extraction was performed: The sample was loaded onto a reverse-phase C18 cartridge (Sep-Pak Waters 12cc 2g) after pre-conditioning with 15 mL of methanol and 30 mL of water. The cartridge was rinsed successively with 50 mL of 90% water/10% acetonitrile and with 10 mL of hexane. The AHLs were then eluted with 20 mL methanol. Fourth, the elution product was evaporated under a nitrogen stream at 50°C. The residue was dissolved in 200 μL of acetonitrile. All solvents were supplemented with 0.1% formic acid.

#### AHL detection by high-pressure liquid chromatography coupled with tandem mass spectrometry

The chromatographic separation of the AHLs was carried out on a Zorbax eclipse XDB-C18 (Agilent Technology, Garches, 92380, France) fitted on an Agilent 1100 HPLC system (91745 Massy, France). The column’s temperature was maintained at 45°C. Five μL of the final extraction product was injected. The mobile phases consisted of (A) water with 0.1% formic acid and (B) acetonitrile with 0.1% formic acid in an 80:20 ratio, respectively. The linear gradient for AHL elution was programmed as follows: after increasing B in A from 20% to 35% for 5 minutes and from 35% to 95% for 15 minutes, the gradient was then kept constant over 10 minutes. Separation was achieved at a flow rate of 0.4 mL/min. Prior to the next injection, the column was equilibrated for 15 minutes. This allowed for the separation of AHLs according to their hydrophobicity depending on the length of the acyl side chain, the nature of the substitution on the third carbon and the presence of unsaturations[20].

Mass spectra were obtained using an API® 2,000 Q-Trap (AB-Sciex, Concord, Ontario, Canada) equipped with a TurboIon electrospray set in the positive mode with nitrogen as the nebuliser gas. The ion source temperature was set at 350°C. Declustering and entrance potentials were set at 60 V and 5,000 V, respectively. Data were acquired by the Analyst® software (version 1.4.2, AB-Sciex) in the multiple reaction monitoring mode. As described by Cataldi et al., identification of an AHL (designated by *m*/*z* of the precursor ion [M+H]^+^) was defined by the presence of the precursor ion and the two possible product ions ([M+H -101]^+^, neutral loss and 102 or lactone moiety) at the same retention time[21] (S1 Fig). The AHL quantification was expressed in nanomole per gram of faeces after calibration with commercially available AHLs (C4-HSL, C7-HSL, 3-OH-C10-HSL, 3-OH-C12-HSL, 3-oxo-C12-HSL, C14-HSL and 3-oxo-C14:1-HSL) and normalisation relative to the internal standard (C6-d3-HSL).

### AHL characterisation

#### High resolution mass spectrometry

Fractions containing AHLs of interest were sampled using a pool of HPLC fractions according to observed retention time and were analysed by high resolution mass spectrometry using an LTQ-Orbitrap XL (ThermoFisher Scientific) with an infusion flow of 3 μL/min. A full scan analysis allowed for obtaining the precise mass of the relevant molecule. Then, the predicted formula was deducted by the integrated software.

#### QS activity

The bacterial biosensor *Agrobacterium tumefasciens* NTL4, kindly provided by Dr P. Williams’ lab (University of Nottingham), was used to assess the QS activity of the identified AHLs. A preculture was grown overnight in Luria Bertani (LB) medium supplemented with gentamycin (30μg/ml) and shaken at 70 rpm at 30°C. AB medium supplemented with 0.5% glucose and thiamine (1mg/l) was inoculated with 1/50th of the overnight preculture’s volume and was grown to late exponential phase (OD_600_ = 0.6, 14 hours). The culture was centrifuged (1700 g for 15 min at 4°C). The bacterial pellet was resuspended in AB medium with a 20-fold concentration, diluted (1/100th) in melted soft agar (0.7%) AB medium with X-gal (final concentration 50 μg/ml)[22] and immediately poured into a petri dish. Twenty microliters of sample and negative (water) and positive controls (standard AHLs) were pipetted into different wells punched into the solidified agar. The plates were incubated in the dark at 30°C for 24–48 hours before a blue coloration around the wells was visible.

#### Search for LuxI and/or LuxR homologues

We tested LuxI (P12747), LuxR (P12746) proteins for similarities, using BLAST (Non-redundant protein sequences, blastp) against the Human Microbiome Project (HMP) "43021[BioProject]". We performed constraint-based multiple alignment using COBALT, and derived the phylogenetic tree with fast minimum evolution algorithm, maximum sequence difference of 0.85 with Grishin distance.

### Microbiota analysis

DNA was extracted as described previously[3] from 200 mg of faeces using the GNOME DNA Kit (MP Biomedicals, Santa Ana, CA) according to the manufacturers' instructions. Real-time qPCR (quantitative polymerase chain reaction) was performed as described previously[23] using an ABI 7,000 Sequence Detection System apparatus with the 7,000 system software v. 1.1 (Applied Biosystems, Foster City, CA). The total bacteria were quantified, including the dominant bacterial groups (*Bacteroides/Prevotella*, *C*. *coccoides*, *C*. *leptum*, *Bifidobacterium*), the subdominant group (*Lactobacillus/Leuconostoc/Pediococcus*) and the specific species *Faecalibacterium prausnitzii* and *Escherichia coli*. Group- and species-specific 16S rRNA-targeted primer sequences purchased from Eurogentec are shown in S1 Table. Due to the quality of DNA extraction among all samples (*n* = 75), 67 (from 25 HS, 21 IBD patients in remission and 21 IBD patients in flare) were analysed through 16S gene sequencing as described previously[24].

### Synthesis of AHL 3-oxo-C12:2

The AHL 3-oxo-C12:2 was synthesised from commercially available 4-pentyn-1-ol (Fluorochem UK, Derbyshire). The alcohol was first protected with chloromethyl methyl ether. The acyl chain was elongated, the desired double bonds were installed by organometal coupling with 1-bromo-2-pentyne, and alkynes were reduced to alkenes. The alcohol was then deprotected and oxidised into carboxylic acid, which was further coupled to Meldrum's acid to afford the active derivative. This derivative was finally reacted with homoserine lactone to obtain the desired molecule (S2 Fig).

### Epithelial cell experiments

#### Caco-2/TC7 cell cultures

Caco-2/TC7 were cultured in 6-well culture plates in high-glucose Dulbecco’s Modified Eagle Medium Glutamax I (GIBCO) supplemented with 20% heat-inactivated foetal calf serum (FCS) (GE Healthcare/ PAA), 1% non-essential amino acids (GIBCO) and 1% penicillin/streptomycin (PAA company) at 37°C in a 10% carbon dioxide/air atmosphere. The culture media were changed every day.

#### Inflammatory response

After 14 days, culture medium was changed for a starvation medium without FCS for 24 hours. Before stimulation, 2-hydroxyquinoline (2HQ), a selective PON inhibitor, was added at the concentration of 100 μM together with 0.1% dimethyl sulfoxide (DMSO) alone or increasing concentrations of 3-oxo-C12 (Sigma-Aldrich®) and 3-oxo-C12:2 in 0.1% DMSO. Caco-2/TC7 cells were stimulated by Interleukin 1 beta (IL-1B) (Sigma-Aldrich®) at 25 ng/mL. After 18 hours, cell supernatants were removed for an Interleukin 8 (IL-8) assay, and cells were washed and scraped into 200 μL Triton 1X. IL-8 concentrations were determined using an enzyme-linked immunosorbent assay (ELISA) (DuoSET Human CxCL8/IL-8, R and D Systems®) and quantified to the total cell-protein content. All experiments were done in duplicate.

#### Permeability assay

Caco-2/TC7 cells were cultured in the same conditions as described above in a 6-well Costar Transwell (3 μm pore size, Sigma-Aldrich®). At confluence, they were switched to asymmetric conditions, i.e., with a medium containing 20% FCS in the basal compartment and serum-free medium in the apical compartment. At day 18, the apical medium was supplemented with 100 μM of PON-inhibitor 2HQ and with DMSO 0.1%, 3-oxo-C12 or 3-oxo-C12:2 (200 μM) for 4 or 20 hours. To assess paracellular permeability, fluorescein isothiocyanate–labelled Dextran 4 kDa (FD4) tracer (TdB consultancy, Uppsala, Sweden®) was added to the apical surface of the cell monolayers to a final concentration of 250 μM. Four hours later, samples of the basal medium were collected and fluorescence was determined with a FLUOstar Omega (BMG Labtech®) calibrated for excitation at 485 nm and emission at 520 nm.

### Statistical analysis

Statistical analyses were performed with the Student’s *t* test for unpaired data and by Wilcoxon’s non-parametric test. Qualitative data were compared within groups using the χ2 test (Jump software, JMP SAS). For searching for the association between the AHL of interest and gut microbiota, differential analyses of bacteria quantity between stool samples were performed using relative quantification (2^-DDCT). For each sample, individual bacteria quantification values were normalised into relative proportion to the total. A correlation test was applied using Pearson’s coefficient.

GraphPad Prism V.6.0 (San Diego, California, USA) was used for all microbiota-sequencing analyses and graph preparation. Results are expressed as mean ± standard error of the mean (SEM), and statistical analyses were performed using the two-tailed, non-parametric Mann–Whitney *U* test or Kruskal–Wallis test with Dunn’s multiple comparison test. The number of observed species and the Shannon and Chao 1 diversity indices were calculated using rarefied data (depth = 2,000 sequences/sample), which are used to characterise species diversity in a community. Statistical significance of sample grouping for beta diversity analysis was performed using the Permanova method (9,999 permutations). Differences with a *p* value < 0.05 were considered significant. Principal component analyses of the Bray Curtis distance with each sample were built and used to assess the variation between experimental groups (beta diversity). Differential analysis was performed using the linear discriminant analysis effect size (LEfSe) pipeline[25]. Multivariate Association with Linear models (MaAsLin), a multivariate statistical framework, was used to find associations between clinical metadata, AHL 3-oxoC12:2 and microbial community abundance. Correlation within microbial taxa abundance data was measured by Spearman’s correlation test.

### Ethics

Local ethics committee of Comite de Protection des Personnes Ile-de-France IV, IRB 445 00003835, Suivitheque study, registration number 2012/05NICB, specifically approved this study.

## Results

### Detection of AHLs in faeces from IBD patients and healthy controls

#### Patients

We collected 75 faecal samples from 49 IBD patients (22 with Crohn’s disease and 27 with ulcerative colitis) during flares (*n* = 24) and remission (*n* = 25) and from 26 HS. The demographic, clinical, biological and therapeutic data of IBD patients and HS are shown in Table 1.

**Table 1:** Patients and controls characteristics.

	HS n = 26	IBD in remission n = 24	Active IBD n = 25
**Sex (male)**	11 (42.3%)	15 (62.5%)	11 (44%)
**Age (years)** (mean+/-SEM)	35.4 +/-2.8	41.5 +/-2.5	35.6 +/- 2.5
**Smokers**	2* (7.7%)	8 (32%)	6 (24%)
**Type of IBD**	—	12 UC (50%)/12 CD	15 UC (60%)/10 CD
**Disease duration (years)** (mean+/-SEM)	—	11.0+/-2.2	4.3+/-1.0*
**Disease location** Montreal classification[26]	—	CD: L1 (n = 3), L2 (n = 2), L3 (n = 7) p (n = 5) UC: E1 (n = 1), E2 (n = 6) E3 (n = 5)	CD: L1 (n = 2), L2 (n = 6), L3 (n = 2) p (n = 6) UC: E1 (n = 1), E2 (n = 7), E3 (n = 7)
**Disease activity scores** (mean+/-SEM)	—	HBI: 1.7 +/-0.3 Mayo: 0.8+/-0.2	HBI: 9.2 +/-1.9** Mayo: 6.7+/-0.3***
**CRP (mg/L)** (mean+/-SEM)	—	4.5+/-1.2	49.1 +/-8.7***
**Hemoglobin (g/dl)** (mean+/-SEM)	—	13.7+/-0.4	11.8+/-0.4*
**Treatment**	—	CS (2), 5ASA(14), IS (9), Biologic (18)	CS (5), 5ASA(12), IS (5), Biologic** (7)

* p < 0.05

**p < 0.005

***p < 0.001: p values vs the IBD group in remission

SEM: standard error of the mean

HS: healthy subject, IBD: inflammatory bowel diseases, UC: ulcerative colitis, CD: Crohn’s disease, HBI: Harvey Bradshaw index, CRP: C-reactive protein, CS: corticosteroids, 5ASA: 5-aminosalicylates, IS: immunosuppressive agent.

#### Identification of N-Acyl homoserine lactones in faeces

**Fig 1:**
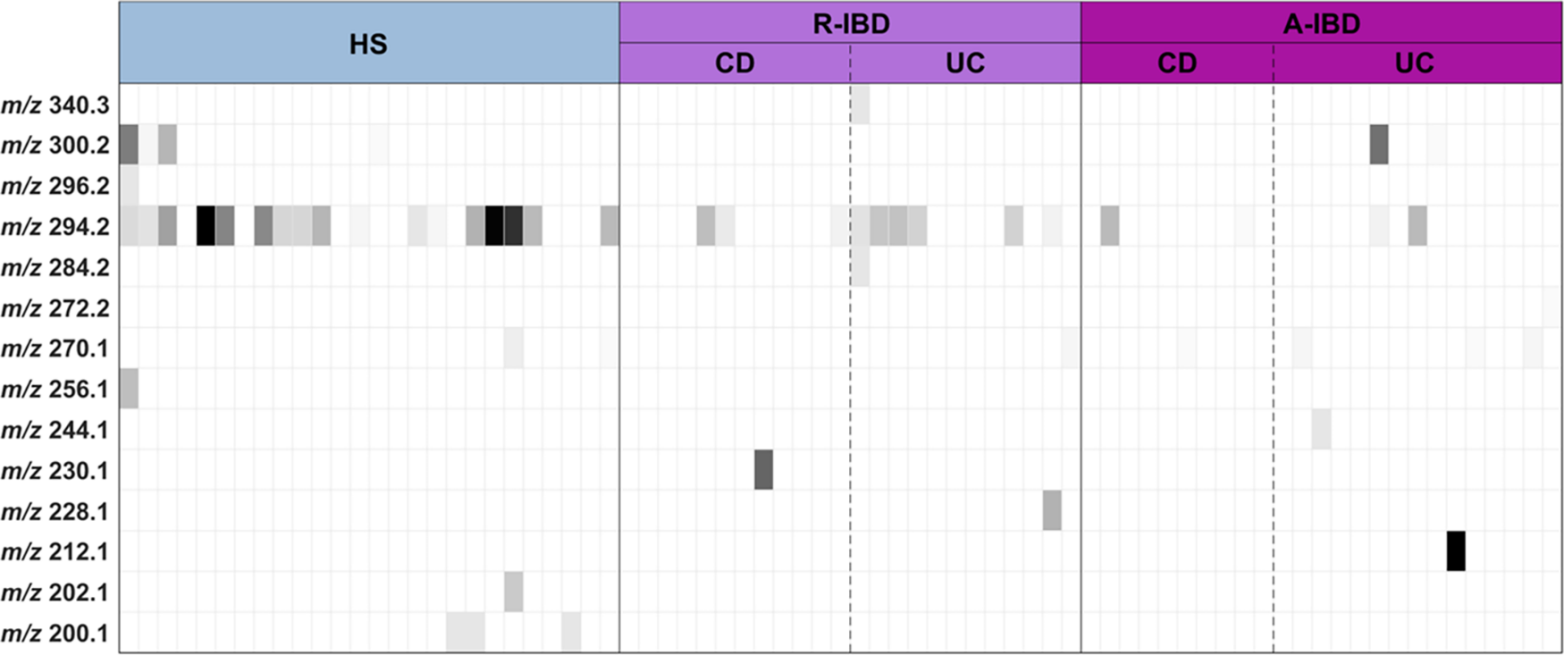
Heatmap of the results of the AHL profile in IBD patients and healthy controls. AHLs are designated by their m/z. The grey colour bar indicates AHL m/z concentrations from none (white) to highest concentration (black). HS: healthy subject, R-IBD: inflammatory bowel disease patients in remission, A-IBD: inflammatory bowel disease patients in flare, UC: ulcerative colitis, CD: Crohn’s disease.

We detected 14 different AHLs from the faecal samples using high-pressure liquid chromatography coupled with tandem mass spectrometry. Each AHL is designated by its *m*/*z* value. The distribution of AHLs among the samples is represented on a heat map (Fig 1A). The AHL at *m*/*z* 294.2 was prominent (30/75 samples, or 40%). All other AHLs were found in less than 10 faecal samples (see Fig 1).

#### Identification of the major faecal AHL: 3-oxo-C12:2

**Fig 2:**
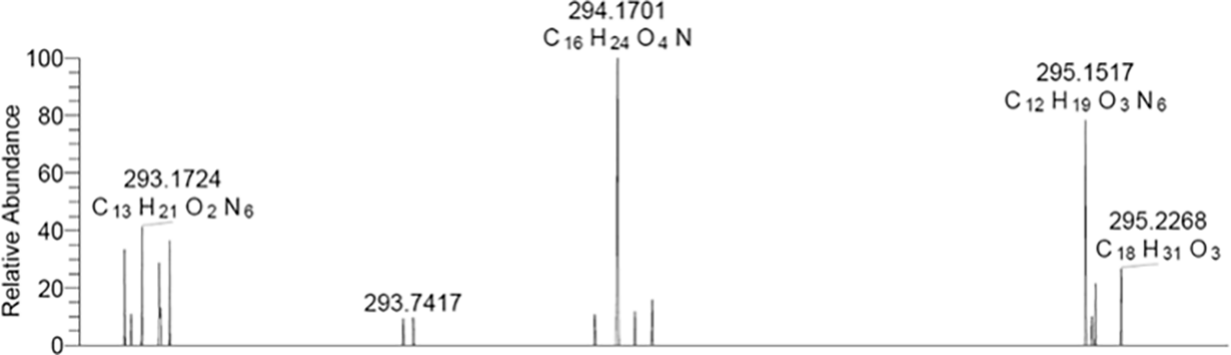
Full scan (80–100) of the fraction containing AHLs at m/z 294.2 using high-resolution mass spectrometry (LTQ-Orbitrap XL) in infusion mode with its precise mass and deducted formula.

Using HPLC, we harvested fractions containing AHL at *m*/*z* 294.2 to obtain its precise mass. Using high-resolution mass spectrometry (LTQ-Orbitrap XL) (Fig 2), we determined the accurate *m*/*z* of this protonated AHL ([M+H]^+^): 294.1700. We deducted its formula (C_16_H_24_O_4_N), which corresponds to unsaturated a N*-*3-oxo-dodecanoyl homoserine lactone with two double bonds (3-oxo-C12:2).

We then tested the pooled fractions containing 3-oxo-C12:2 on the NTL4 biosensor. The concentrated fraction (X5) tested positive (S3 Fig), confirming the QS activity of this AHL.

#### Distribution of 3-oxo-C12:2 in IBD patients

**Fig 3:**
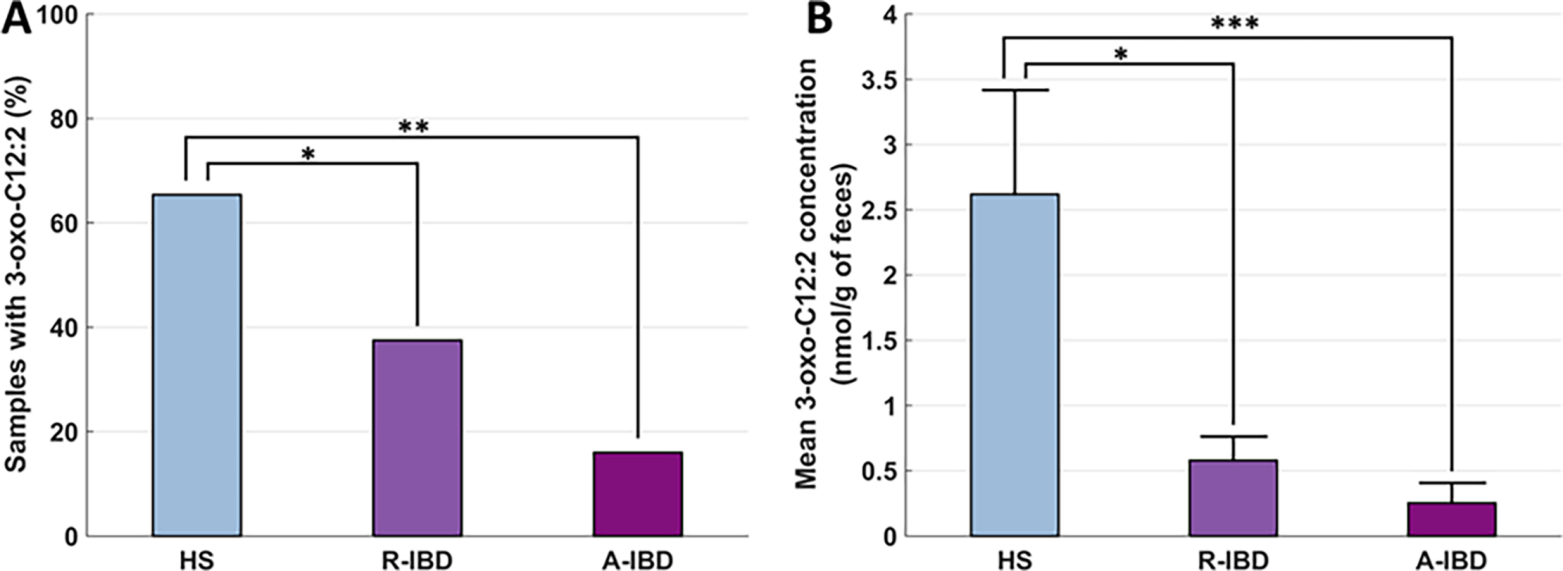
Bar graph of AHL 3-oxo-C12:2 distribution and concentration among healthy subjects (HS) and IBD in remission (R-IBD) and in flare (A-IBD). A: proportion of samples with AHL 3-oxo-C12:2. B: mean 3-oxo-C12:2 faecal concentration. * p < 0.05 **p < 0.005 ***p < 0.0005.

The 3-oxo-C12:2 AHL was found significantly more often in HS (17/26, 65.4%) than in IBD patients in flare (4/25, 16%) and IBD patients in remission (9/24, 37.5%) (*p* = 0.001) (Fig 3A). Furthermore, 3-oxo-C12:2 faecal concentrations were significantly higher in HS (2.62 ± 0.80 nmol/g of faeces) compared to IBD patients in remission (0.58 ± 0.18 nmol/g of faeces, *p* = 0.014) and in flare (0.25 ± 0.15 nmol/g of faeces, *p* = 0.0002) (Fig 3B).

### Presence of 3-oxo-C12:2 is associated with gut microbiota profiles

#### Faecal microbiota composition

**Fig 4:**
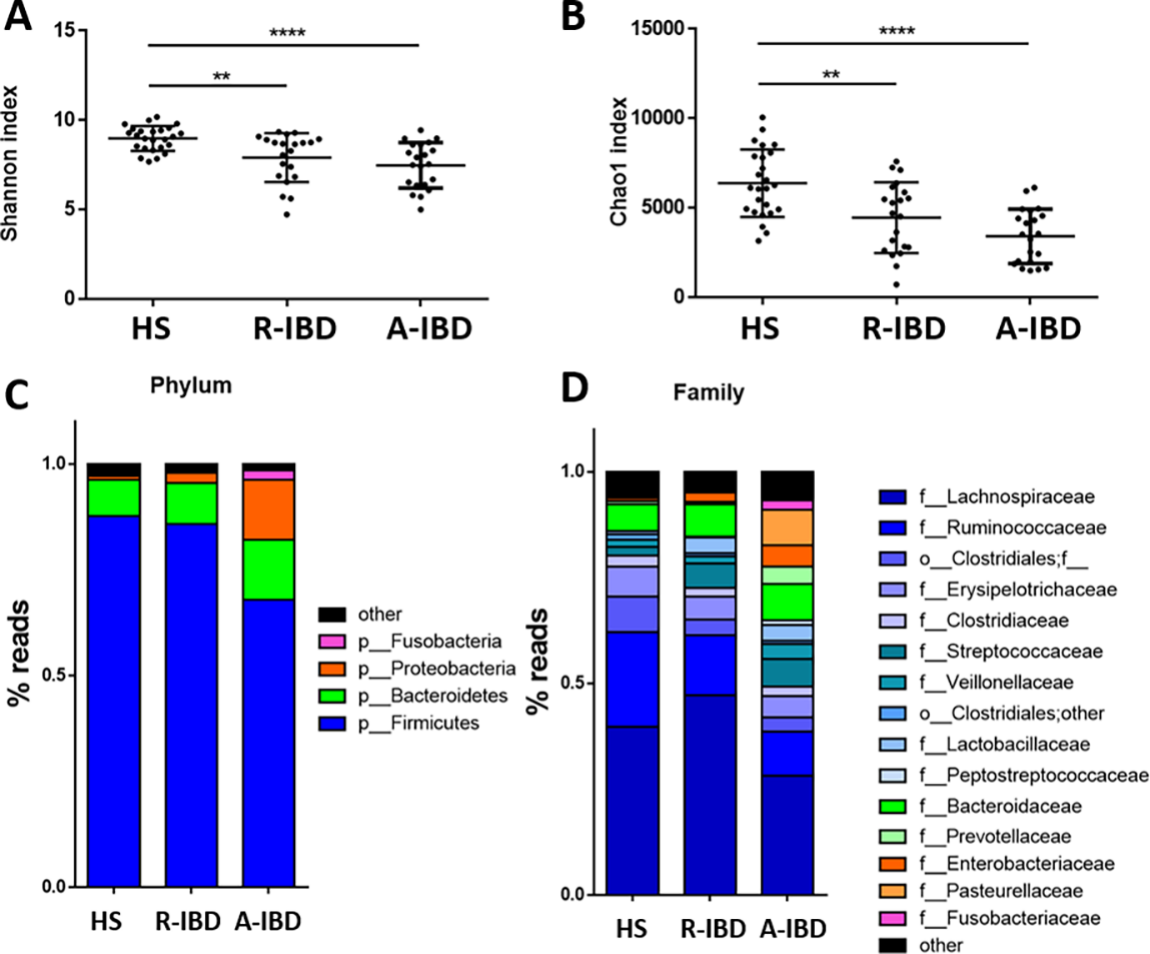
**Bacterial diversity and composition among healthy subjects (HS), IBD in remission (R-IBD) and in flare (A-IBD):** alpha diversity Shannon index (A) and Chao 1 index (B) and composition at a phyla scale (C) and at family scale (D).

**Fig 5:**
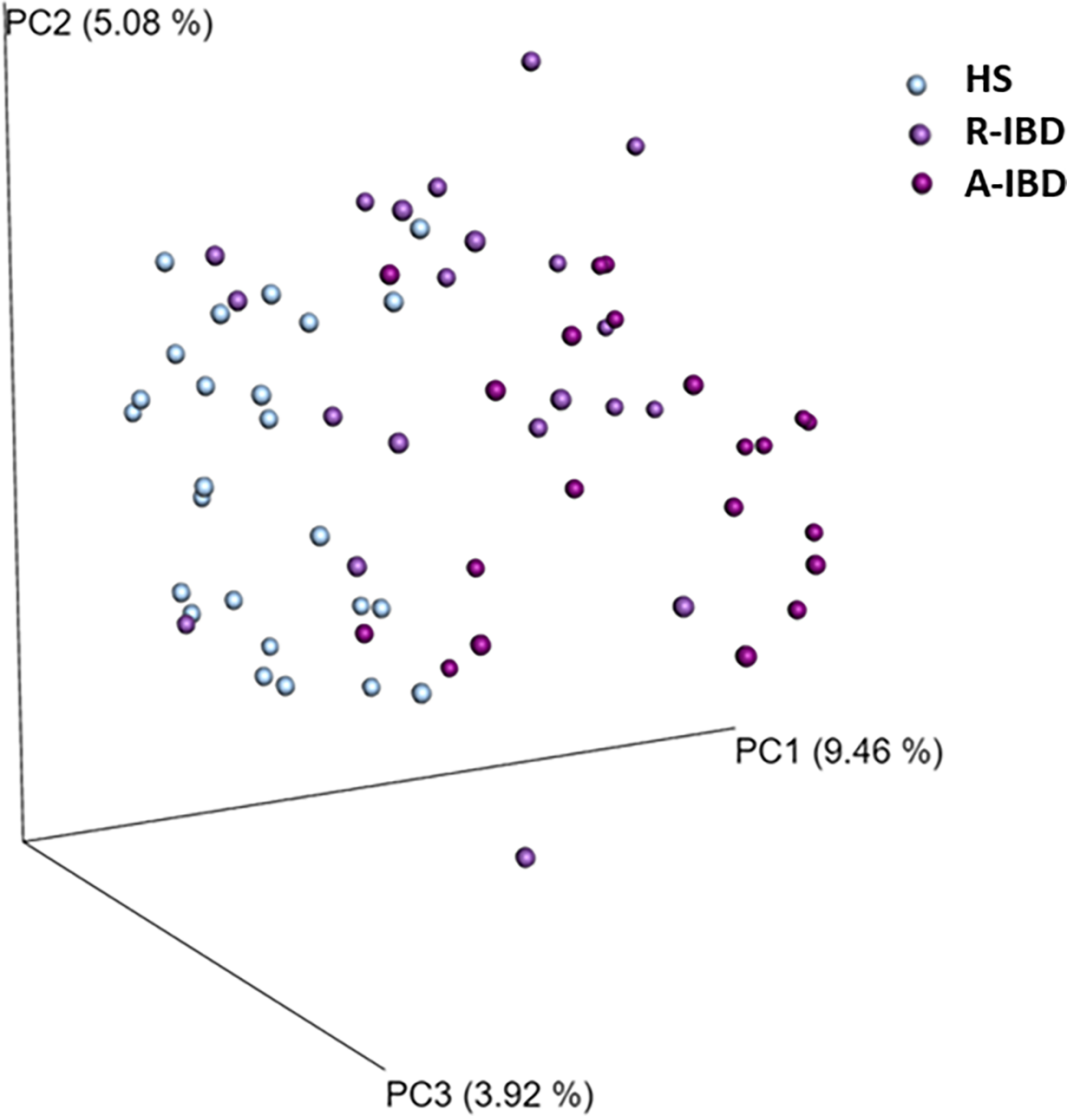
Beta diversity according to Bray Curtis index between healthy subjects (HS) and IBD in remission (R-IBD) and in flare (A-IBD).

We observed a loss in the alpha diversity of gut microbiota in IBD patients compared to HS (Fig 4A and 4B). Besides a loss in biodiversity, changes in bacterial composition in IBD patients during remission and flare were comparable with previously described dysbiosis (Fig 4C and 4D). They were characterised by a loss in Firmicutes (especially Lachnospiraceae and Ruminococcaceae) and an expansion in Proteobacteria. Principal component analysis of microbiota sequencing data showed a different repartition in HS compared to IBD patients in remission and in flare (Fig 5).

Real-time qPCR analysis also showed dysbiosis among IBD patients, especially IBD patients in flare (S4 Fig). There were significantly lower counts of *Clostridium coccoides* and *Faecalibacterium prausnitzii* and significantly higher concentrations of *Bacteroides* and *Escherichia coli* in IBD patients in flare compared to HS. The *Clostridium leptum* group was significantly less represented in IBD patients in flare and in remission compared to HS.

#### 3-oxo-C12:2 correlation with normobiosis

**Fig 6:**
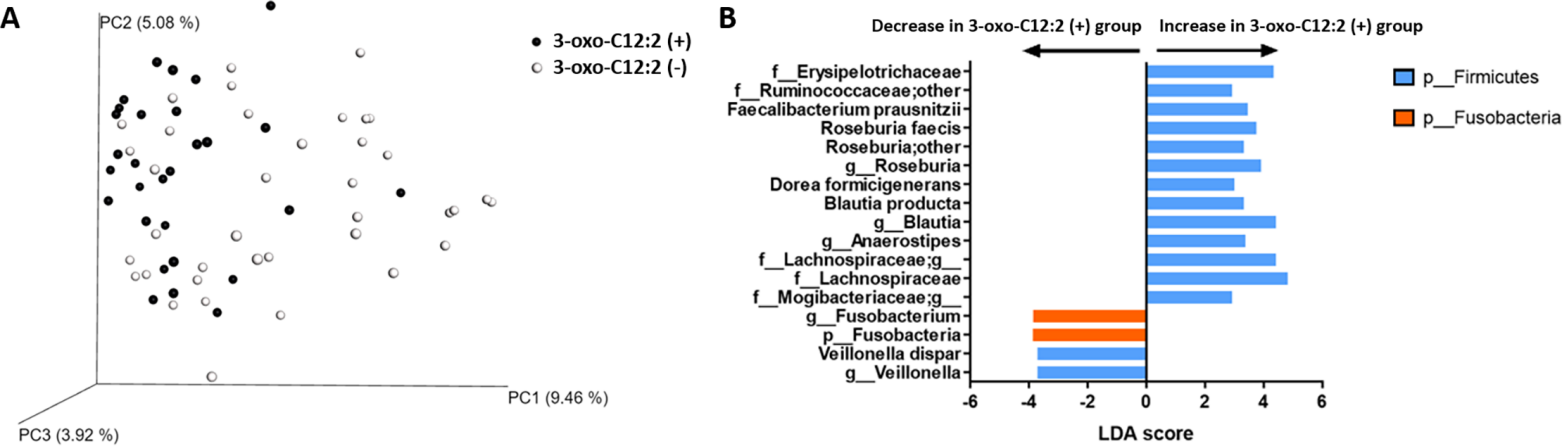
Bacterial composition among faecal samples according to AHL 3-oxo-C12:2 detection (3-oxo-C12:2 [+] group) or AHL 3-oxo-C12:2 absence (3-oxo-C12:2 [–] group). A: Beta diversity according to Bray Curtis index between the two groups. B: Bacterial taxa that were differentially represented in the 3-oxo-C12:2 (+) group with statistical levels of significance according to linear discriminant analysis (LDA score > 2). Taxa were identified at the order, family, gender or species level and colour-coded according to their phylum.

**Fig 7:**
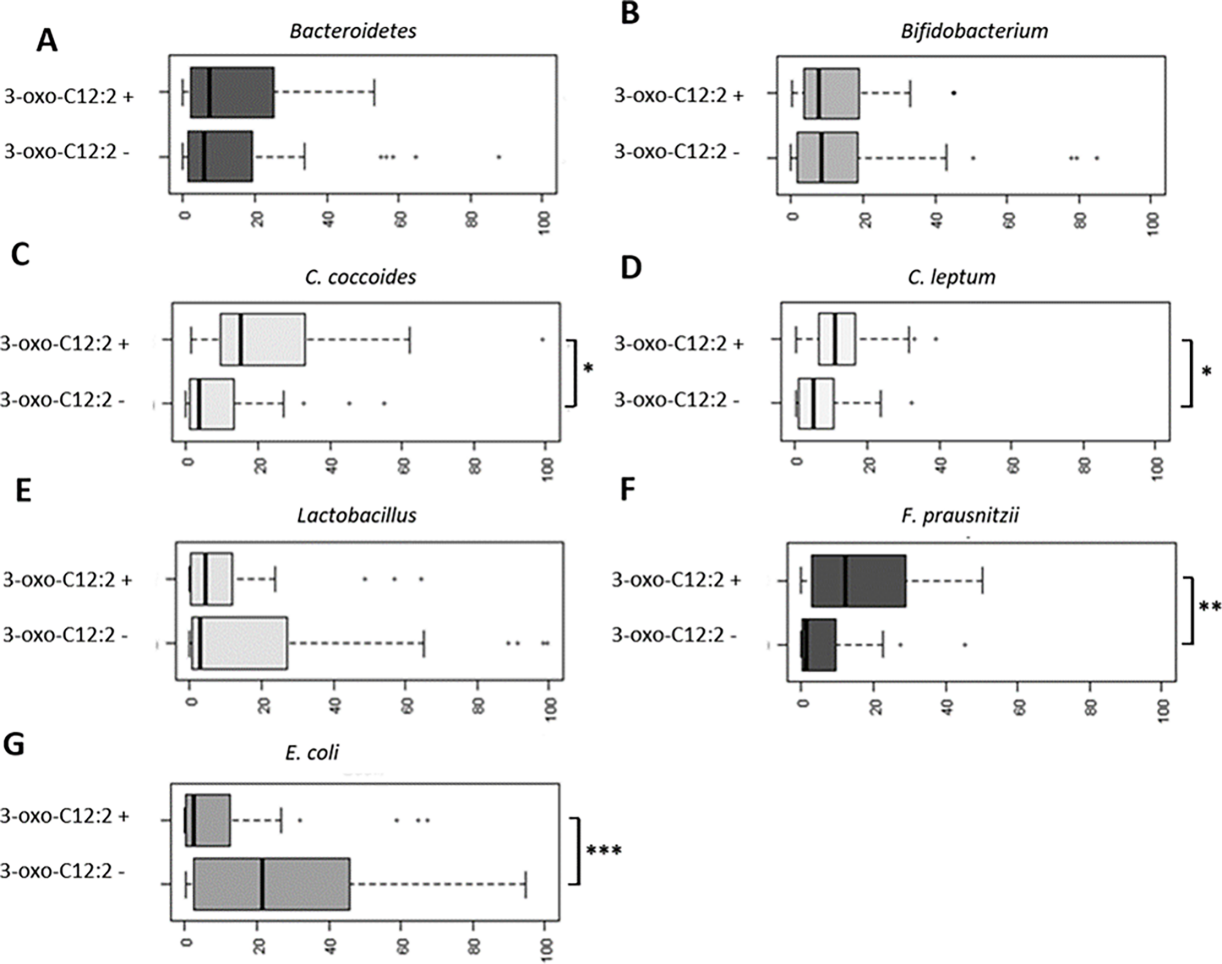
Relative quantification (2^-DDCT) of gut microbiota principal bacterial groups and species. * p < 0.05 **p < 0.005 *** p < 0.001.

We compared faecal microbiota composition between faecal samples in which 3-oxo-C12:2 was present and those in which it was not detected. Principal component analysis of microbiota sequencing data showed a different distribution (Fig 6A). As shown through LEfSe analysis, the main contributors to discriminating 3-oxo-C12:2–associated bacterial groups were Erysipelotrichaceae, Ruminococcaceae, *Roseburia*, *Blautia*, Lachnospiraceae and *Faecalibacterium prausnitzii* (Fig 6B). Altogether, our results indicate that 3-oxo-C12:2 correlates positively with normobiosis. To validate this hypothesis, we performed correlation tests first using 16S sequencing and then qPCR data. From the sequencing data, 3-oxo-C12:2 correlated positively with Lachnospiraceae (such as *Anaerostipes*, *Roseburia* and *Blautia*), Coriobacteriaceae and Ruminococcaceae (such as *Faecalibacterium prausnitzii*). It correlated negatively with Fusobacteriaceae and Veillonellaceae (S2 Table). From real-time qPCR data, which gives targeted and more quantitative results, the presence of 3-oxo-C12:2 was associated with significantly higher counts of Firmicutes (the *C*. *coccoides* and *C*. *leptum* groups and especially *F*. *prausnitzii*) and lower counts of *E*. *coli* as shown in Fig 7.

Finally, we performed a search for LuxI and/or LuxR homologues within the HMP database to trying to identify bacterial species potentially responsible for 3-oxo-C12:2 synthesis. As expected, some proteobacteria from gut ecosystem shared LuxI/R homologues. However, we did not found any LuxI and/or LuxR homologues in Firmicutes (S5 and S6 Figs).

### 3-oxo-C12:2 exerts a protective effect on the human gut epithelial Caco-2/TC7 cells

#### Effect of 3-oxo-C12:2 on inflammatory response in Caco-2/TC7 cells

**Fig 8:**
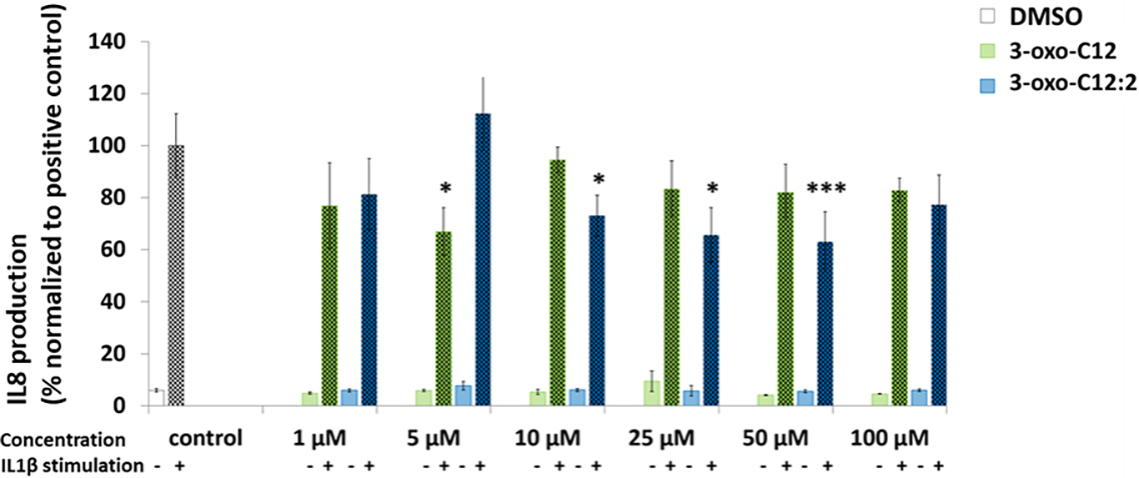
IL-8 (normalized ratio) secretion by Caco-2/TC7 cells before and after IL1β stimulation with increasing concentrations of AHLs 3-oxo-C12 and 3-oxo-C12:2 compared to control (DMSO 0.1%). *p < 0.05 ***p < 0.001.

We observed a significant decrease in IL-1β–induced IL-8 secretion when adding 3-oxo-C12:2 at 10, 25 and 50 μM as well as 3-oxo-C12 at 5 μM as compared to the control (Fig 8).

#### Effect of 3-oxo-C12:2 on paracellular permeability in Caco-2/TC7 cells

**Fig 9:**
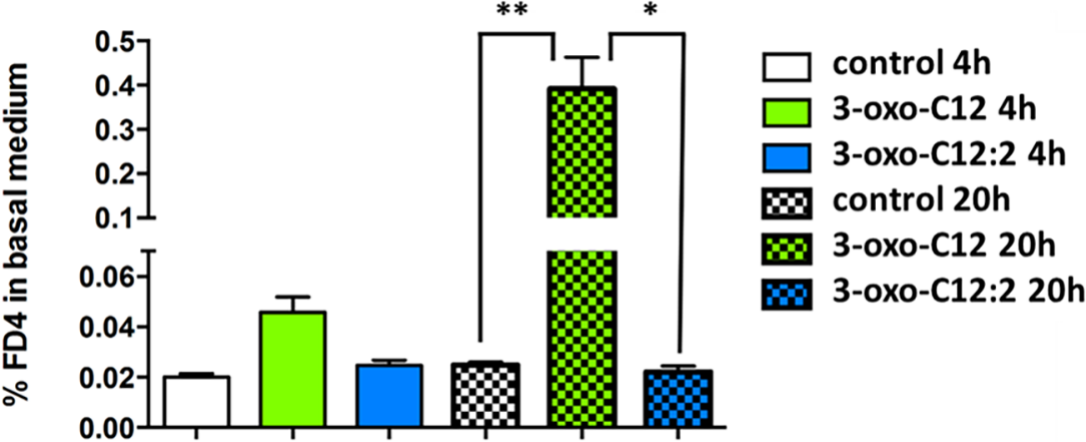
Proportion of FD4-FITC in the basal medium after 4 hours on Caco-2/TC7 cells cultured in transwell. Cells were pre-exposed with AHLs 3-oxo-C12 and 3-oxo-C12:2 at 200 μM for 4 and 20 hours. *p < 0.05 **p < 0.01.

3-oxo-C12:2 showed no modification of paracellular permeability after 4 or 20 hours of exposure. On the contrary, 3-oxo-C12 increased paracellular permeability after 4 hours of exposure (X2, non-significant) and 20 hours exposure (X15, *p* < 0.01) compared to the control (Fig 9).

## Discussion

In this study, we identified for the first time AHLs in human faeces and observed that the AHL 3-oxo-C12:2, which has never been described so far, was prominent in this ecosystem. This AHL correlated with normobiosis and was lost in IBD patients, especially those in flare. Finally, we observed that this molecule exerts a protective effect on gut epithelial cells; i.e., an anti-inflammatory effect with no increase in paracellular permeability.

QS involving AHLs has been described in various ecosystems[27,28] and in gut pathogens[29] but never in human faeces. In 2013, Swearingen et al. addressed the question: “Are there Acyl-homoserine lactones within mammalian intestines?”[19] They ventured the hypothesis that AHLs were produced within the complex microbial communities of the human intestinal tract even though they had never been detected. After developing an original extraction method specifically for faecal samples, the sensitivity of mass spectrometry enabled us to confirm this hypothesis. The first and most challenging step of this work was to determine the best extraction method based on two major concerns: the first was to catch the largest rank of AHLs that exhibit variable hydrophobicity, and the second was to extract a small molecule in a complex and solid sample with a tremendous number of molecules. We defined a two-step extraction, beginning with solid-liquid extraction using a Soxhlet extractor, followed by solid phase extraction, taking into consideration the amphiphilic properties of AHLs. We thus identified a prominent AHL in the human gut microbiota, and high-resolution mass spectrometry allowed us to determine its precise mass and therefore its chemical formula: 3-oxo-C12:2. We discovered a never before identified AHL, 3-oxo-C12:2, which is prominent in the human gut microbiota. Still, some limitations can be raised about other AHLs present in gut ecosystem, which could have not been detected with our technique because of a lower concentration than 3-oxo-C12:2 and/or because of a lower yield of the extraction method. In fact, the yield of the extraction method differs according to AHL chemical structure. Also, some uncertainty persists about the molecule 3-oxo-C12:2 as we could not determine the position and the isomer (cis or trans) of the two double bounds. To go further, we need to display a larger quantity of 3-oxo-C12:2 and go beyond the limits of current technology. For example, one can determine by gas chromatography coupled with tandem mass spectrometry after derivation the position of the double bound in the acyl side chain by the DMDS (dimethyl disulfide) method[30]. These kinds of experiments remain challenging. Even though some other AHLs may have been missed by our extraction technique, the detection of 3-oxo-C12:2 in the gut microbiota from human samples is relevant. Indeed, its concentration in a cohort of IBD patients and healthy controls was associated with a healthy state and normobiosis. Thanks to a translational strategy built on a well-phenotyped IBD cohort and the expertise of gut microbiota analysis, we have brought forward an original result on the gut ecosystem. Moreover, considering that AHLs production is linked to the quorum state of microbial communities, we believe that this type of molecule is informative when studying the functional consequences of microbiota imbalance.

Our exploratory study raises some fundamental questions about the place of the 3-oxo-C12:2 AHL in the human gut microbiota: Which bacterial species or family can synthesise it? Which ones recognise it? Could it influence the balance of gut microbiota and in which way? The analysis of microbiota sequencing data began to address these questions. It showed correlations between 3-oxo-C12:2 and some bacterial species or families (mostly Firmicutes), but none of them were strong enough to identify a bacterial candidate for 3-oxo-C12:2 synthesis nor a bacterial target. The positive correlation between 3-oxo-C12:2 and Firmicutes rather suggests that 3-oxo-C12:2 is a marker of normobiosis than that it is directly synthetized by Firmicutes. A possible explanation is that the 3-oxo-C12:2 AHL is a final product of a trophic chain involving several different bacterial species from gut microbiota. A simple LuxI-/LuxR-type of quorum sensing as described in a single bacterial species population is unlikely to happen in a complex ecosystem such as the gut microbiota. Moreover, there was no evidence for the presence of LuxI AHL synthase within the Firmicutes. As expected, some proteobacteria from gut ecosystem shared LuxI/R homologues. Thus, we think that is likely that unknown AHL synthase genes with low or no homology with LuxI genes[28,31] encoded for the 3-oxo-C12:2 AHL synthases. Besides, the relative complexity of the 3-oxo-C12:2 AHL (long acyl side chain, two double bonds) is compatible with a several-step synthesis. A thorough bioinformatic analysis of AHL synthase, AHL receptors and desaturase genes in the gut microbiota genome could be used as a strategy to identify bacterial candidates. This experimental plan has been used in the marine ecosystem[28]. It is possible that 3-oxoC12:2 AHL is synthesized by Gram (-) bacteria, directly or indirectly following a series of bio-modifications (enzymatic or non-enzymatic ones) including bacterial decarboxylases (carboxy-lyases), desaturases or other reactions. Although the intestinal tract is considered to be the most important reservoir of *Pseudomonas aeruginosa*[32], this bacteria species belongs to the gammaproteobacteria phylum which is not a major one in human gut microbiota. Thus, it is unlikely that 3-oxoC12:2 AHL could come from desaturation of 3-oxoC12. Administration of 3-oxo-C12:2 AHL in a murine model could help identify the potential role of this AHL in regulating microbial communities. Our hypothesis is that quorum sensing and, more precisely, the lack of 3-oxo-C12:2 could be a factor influencing the persistence of dysbiosis in IBD.

Beyond these ecological considerations, the discovery of 3-oxo-C12:2 as a prominent AHL of the gut ecosystem invited us to test its biological role in the host, showing a protective effect of 3-oxo-C12:2 on gut epithelial cells. Pragmatically, we decided to synthesise 3-oxo-C12:2 with the most likely position of double bounds. We felt confident that the anti-inflammatory effect of 3-oxo-C12:2 could be shown. In fact, the structurally close AHL 3-oxo-C12 produced by *Pseudomonas aeruginosa* has been previously described to have an anti-inflammatory effect on several cell types different from epithelial cells[33–35]. Furthermore, it has also been shown that AHLs with a chemical structure close to 3-oxo-C12-AHL (long acyl chain, oxo substitution, intact lactone ring moiety, etc.) present a similar anti-inflammatory effect[36]. We found an anti-inflammatory effect of both 3-oxo-C12 and 3-oxo-C12:2 on Caco-2 cells without visible cytotoxicity. We took into account PON expression in this cell type, and therefore we used a selective PON-inhibitor to assess the anti-inflammatory effect and thus preserved the lactone ring moiety. Note that the integrity of the lactone ring moiety is fundamental for the AHL anti-inflammatory effect[36]. 3-oxo-C12:2 exerts an anti-inflammatory effect at a higher concentration than 3-oxo-C12 but in a wider range of concentrations. This suggests that the two double bonds contribute to an increased stability of this AHL by favouring folding. Although the immunomodulatory effect of 3-oxo-C12 has been well described, the AHL receptors and molecular partners in host cells still need to be precisely identified. It has been suggested that this effect results from the interaction of AHLs with peroxisome proliferator-activated receptor (PPAR) within the host cell[37].

Exploring the other potential effects of AHLs on Caco-2 cells, we confirmed that 3-oxo-C12 increases paracellular permeability[38,39]. Indeed, this AHL secreted by a pathogen is a part of the invasion strategy of the host. The most striking result is that the structurally close but unsaturated 3-oxo-C12:2 does not modify paracellular permeability. On one hand, we have a quorum sensing molecule from a pathogen that increases para-cellular permeability and on the other hand we described a prominent quorum sensing molecule from commensal microbiome that did not impair this key parameter of the intestinal barrier function. This finding reinforces the interest in studying a molecule that is likely to exert protective effects on gut mucosa especially in therapeutic perspective in IBD where increase in intestinal permeability is common. Indeed, Increased epithelial permeability, also called ‘leaky gut’[40], is a very early phenomenon in IBD pathophysiology. It has been described in first-degree relatives without clinical symptoms[41], and it could be very appealing to explore if 3-oxo-C12:2 could partly restore the intestinal epithelial barrier.

These results pave the way to the use of 3-oxo-C12:2 as an ecological immunomodulator. One can speculate that this prominent AHL could both restore normobiosis and have an anti-inflammatory effect without increasing permeability in the case of IBD-associated dysbiosis. Manipulation of gut microbiota in order to control inflammation in IBD has been strongly investigated in the past few years. This has led to the successful use of faecal microbiota transplantation in IBD patients, especially in UC[42,43]. This drastic method is not completely satisfactory and exposes patients to potential long-term risks[44]. Besides, antibiotics have been shown to be efficient in IBD in specific situations (e.g., post-operative recurrence, pouchitis, perianal lesions). However, it raises concerns about antibiotic resistance, and antibiotics should not be used in long-lasting diseases such as IBD. Probiotics have failed to reach a level of significant efficacy to be used for IBD[45,46]. In this setting, future developments will probably come from natural products from gut microbiota to control gut inflammation. In this therapeutic perspective, 3-oxo-C12:2, through its dual effect on host and microbiota, appears to be a good candidate. As a proof of concept, our results constitute the first step towards an ecological, therapeutic approach to IBD.

## Conclusion

In conclusion, using state-of-the-art mass spectrometry in a targeted strategy, we were able to investigate quorum-sensing molecules, such as AHLs, from the human gut microbiota for the first time. We discovered a new and prominent AHL in gut microbiota, 3-oxo-C12:2, which exhibits a protective effect on gut epithelial cells. These innovative results contribute to improving our knowledge of the interactions between the human gut microbiota and the host. Through a translational approach, we explored the potential effects of the loss of 3-oxo-C12:2 in IBD-associated dysbiosis and in gut epithelial inflammation. In the field of ecological therapeutic approaches to treating IBD, our results should be considered as a cornerstone for a new concept to control gut inflammation and to manipulate gut microbiota.

## Supporting information

S1 Table Group and species-specific 16S rRNA-targeted primers and their sequences. (DOCX)

S2 Table Correlations between 3-oxo-C12:2 concentration and bacterial taxa from 16S sequencing. Taxa: In the first column, taxa were identified at the order, family, gender or species level. Spearman ρ: Spearman's rank correlation coefficient (non-parametric Spearman’s test) between 3-oxo-C12:2 concentration and bacterial taxa from 16S sequencing. For each correlation coefficient, the corresponding p-value appears in the third column.
(DOCX)

S1 Fig MS-MS fragmentation of an AHL with the two product ions ([M+H -101]+ and 102) obtained from the precursor ion [M+H]+. (TIF)

S2 Fig Steps of 3-oxo-C12:2 synthesis from 4-pentyn-1-ol. (TIF)

S3 Fig Results of NTL4 biosensor with AHL at m/z 294.2. 1: control (water) 2: C7 5μM 3: 3-oxo-C12 5μM, HPLC fraction with AHL at m/z 294.2 with different concentrations : 4 : X1/10, 5 : X1/2, 6 : X1, 7 : X2.5, 8 : X5.
(TIF)

S4 Fig Quantification of dominant and subdominant bacteria in the fecal microbiota from IBD patients in remission (R-IBD) and flare (A-IBD) and healthy controls (HS). A: All bacteria in Log10 CFU Equivalent/g of feces (+/-SEM); B, C, D, E, F, G, H : ΔΔCT (+/-SEM) qPCR of dominant and subdominant bacterial groups and specific species in feces. Thin bars indicates significant comparisons.
(TIF)

S5 Fig Phylogenetic tree of LuxI gene within the Human Microbiome project database. Phylogenetic tree was contructed with fast minimum evolution algorithm and maximum sequence difference of 0.85 with Grishin distance highlithed sequence corresponds to LuxI original sequence. Blast names color map : blue : g-proteobacteria; light blue: b-proteobacteria; pink : proteobacteria; red: bacteria; grey : a-proteobacteria; beige : d-proteobacteria; green : other sequences.
(TIFF)

S6 Fig Phylogenetic tree of LuxR gene within the Human Microbiome project database. Phylogenetic tree was constructed with fast minimum evolution algorithm and maximum sequence difference of 0.85 with Grishin distance highlithed sequence corresponds to LuxR original sequence. Blast names color map : blue : g-proteobacteria; light blue: b-proteobacteria; pink : proteobacteria; red: bacteria; grey : a-proteobacteria; beige : d-proteobacteria; green : other sequences.
(TIFF)
