# Supplementary material for: Inter-kingdom effect on epithelial cells of the N-Acyl homoserine lactone 3-oxo-C12:2, a major quorum-sensing molecule from gut microbiota
Source: PLoS One. 2018 Aug 29;13(8):e0202587. doi: 10.1371/journal.pone.0202587 (PMC6114859; doi:10.1371/journal.pone.0202587)
Supplement: S1 Table — (DOCX) [file pone.0202587.s001.docx]

S1 Table. Group and species-specific 16S rRNA-targeted primers and their sequences

| Target | Primer | Sequence 5’-3’ |
| --- | --- | --- |
| All bacteria | F_Bact 1369 | CGG TGA ATA CGT TCC CGG |
|  | R_Prok 1492 | TAC GGC TAC CTT GTT ACG ACT T |
| *Bacteroides* | Bacter 11 | CCT WCG ATG GAT AGG GGT T |
|  | Bacter 08 | CAC GCT ACT TGG CTG GTT CAG |
| *Coccoides* | Ccoc 07 | GAC GCC GCG TGA AGG A |
|  | Ccoc 14 | AGC CCC AGC CTT TCA CAT C |
| *Clostridium leptum* | Clept 09 | CCT TCC GTG CCG SAG TTA |
|  | Clept 08 | GAA TTA AAC CAC ATA CTC CAC TGC TT |
| *Bifidobacterium* | F_bifid 09c | CGG GTGAGT AAT GCG TGA CC |
|  | R_bifid 06 | TGA TAG GAC GCG ACC CCA |
| *Lactobacillus* | Lacto 04 | CGC CAC TGG TGT TCY TCC ATA |
|  | Lacto 05 | AGC AGT AGG GAA TCT TCC A |
| *Faealibacterium prausnitzii* | F.Prau 07 | CCA TGA ATT GCC TTC AAA ACT GTT |
|  | F.Prau 02 | GAG CCT CAG CGT CAG TTG GT |
| *Escherichia coli* | E. Coli F | CAT GCC GCG TGT ATG AAG AA |
|  | E. Coli R | CGG GTA ACG TCA ATG ATG AGC AAA |
