# Supplementary material for: Inter-kingdom effect on epithelial cells of the N-Acyl homoserine lactone 3-oxo-C12:2, a major quorum-sensing molecule from gut microbiota
Source: PLoS One. 2018 Aug 29;13(8):e0202587. doi: 10.1371/journal.pone.0202587 (PMC6114859; doi:10.1371/journal.pone.0202587)
Supplement: S2 Table — Taxa: In the first column, taxa were identified at the order, family, gender or species level. Spearman ρ: Spearman's rank correlation coefficient (non-parametric Spearman’s test) between 3-oxo-C12:2 concentration and bacterial taxa from 16S sequencing. For each correlation coefficient, the corresponding p-value appears in the third column. (DOCX) [file pone.0202587.s002.docx]

S2 Table. Correlations between 3-oxo-C12:2 concentration and bacterial taxa from 16S sequencing

| Taxa | Spearman ρ | p value |
| --- | --- | --- |
| p__Firmicutes;c__Clostridia;o__Clostridiales;f__Lachnospiraceae;g__Anaerostipes;s__ | 0,48927007 | 0.001 |
| p__Firmicutes;c__Clostridia;o__Clostridiales;f__Lachnospiraceae;g__Roseburia;Other | 0,42245372 | 0.005 |
| p__Actinobacteria;c__Coriobacteriia;o__Coriobacteriales;f__Coriobacteriaceae;g__;s__ | 0,3882074 | 0.01 |
| p__Firmicutes;c__Clostridia;o__Clostridiales;f__Lachnospiraceae;g__;s__ | 0,3751996 | 0.01 |
| p__Firmicutes;c__Clostridia;o__Clostridiales;f__Lachnospiraceae;g__Roseburia;s__faecis | 0,3720511 | 0.01 |
| p__Firmicutes;c__Clostridia;o__Clostridiales;f__Ruminococcaceae;g__Faecalibacterium;s__prausnitzii | 0,35554197 | 0.02 |
| p__Fusobacteria;c__Fusobacteriia;o__Fusobacteriales;f__Fusobacteriaceae;g__Fusobacterium;s__ | -0,3554251 | 0.02 |
| p__Firmicutes;c__Clostridia;o__Clostridiales;f__[Mogibacteriaceae];g__;s__ | 0,35476241 | 0.02 |
| p__Firmicutes;c__Clostridia;o__Clostridiales;f__Lachnospiraceae;g__Blautia;s__ | 0,35101284 | 0.02 |
| p__Firmicutes;c__Clostridia;o__Clostridiales;f__Ruminococcaceae;Other;Other | 0,34656497 | 0.02 |
| p__Firmicutes;c__Clostridia;o__Clostridiales;f__Lachnospiraceae;g__Dorea;s__formicigenerans | 0,34594418 | 0.02 |
| p__Actinobacteria;c__Actinobacteria;o__Actinomycetales;f__Corynebacteriaceae;g__Corynebacterium;s__durum | 0,33126643 | 0.03 |
| p__Firmicutes;c__Clostridia;o__Clostridiales;f__Veillonellaceae;g__Veillonella;s__dispar | -0,3278163 | 0.03 |
| p__Firmicutes;c__Erysipelotrichi;o__Erysipelotrichales;f__Erysipelotrichaceae;g__;s__ | 0,31502506 | 0.04 |
