# Supplementary figures and images for: Inter-kingdom effect on epithelial cells of the N-Acyl homoserine lactone 3-oxo-C12:2, a major quorum-sensing molecule from gut microbiota
Source: PLoS One. 2018 Aug 29;13(8):e0202587. doi: 10.1371/journal.pone.0202587 (PMC6114859; doi:10.1371/journal.pone.0202587)

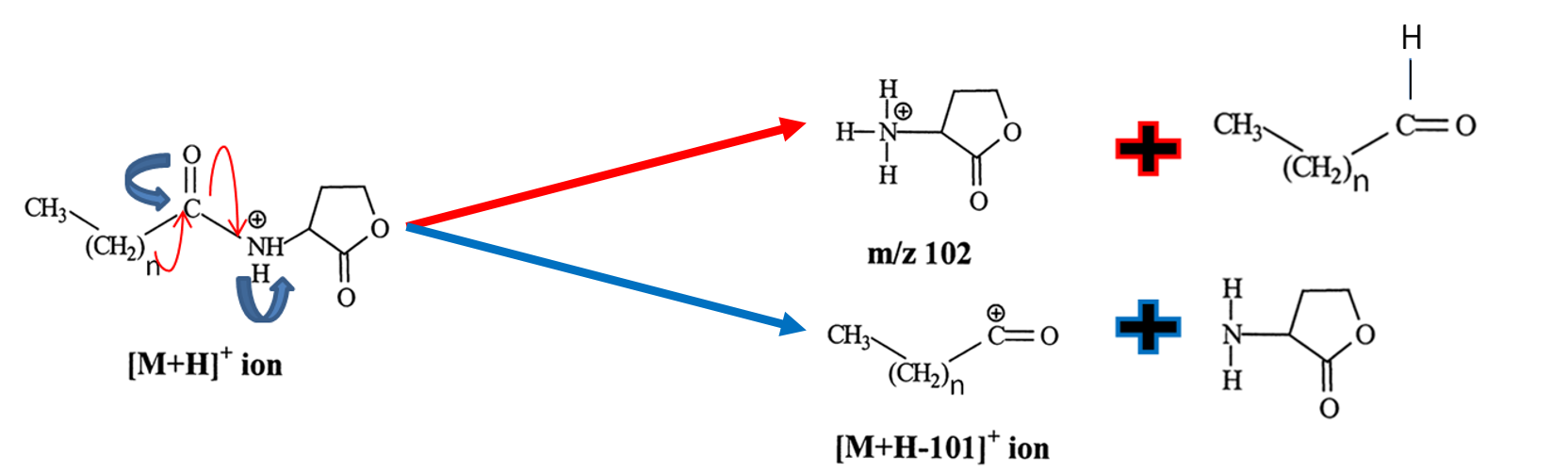

Supplement: S1 Fig — (TIF) [file pone.0202587.s003.tif]

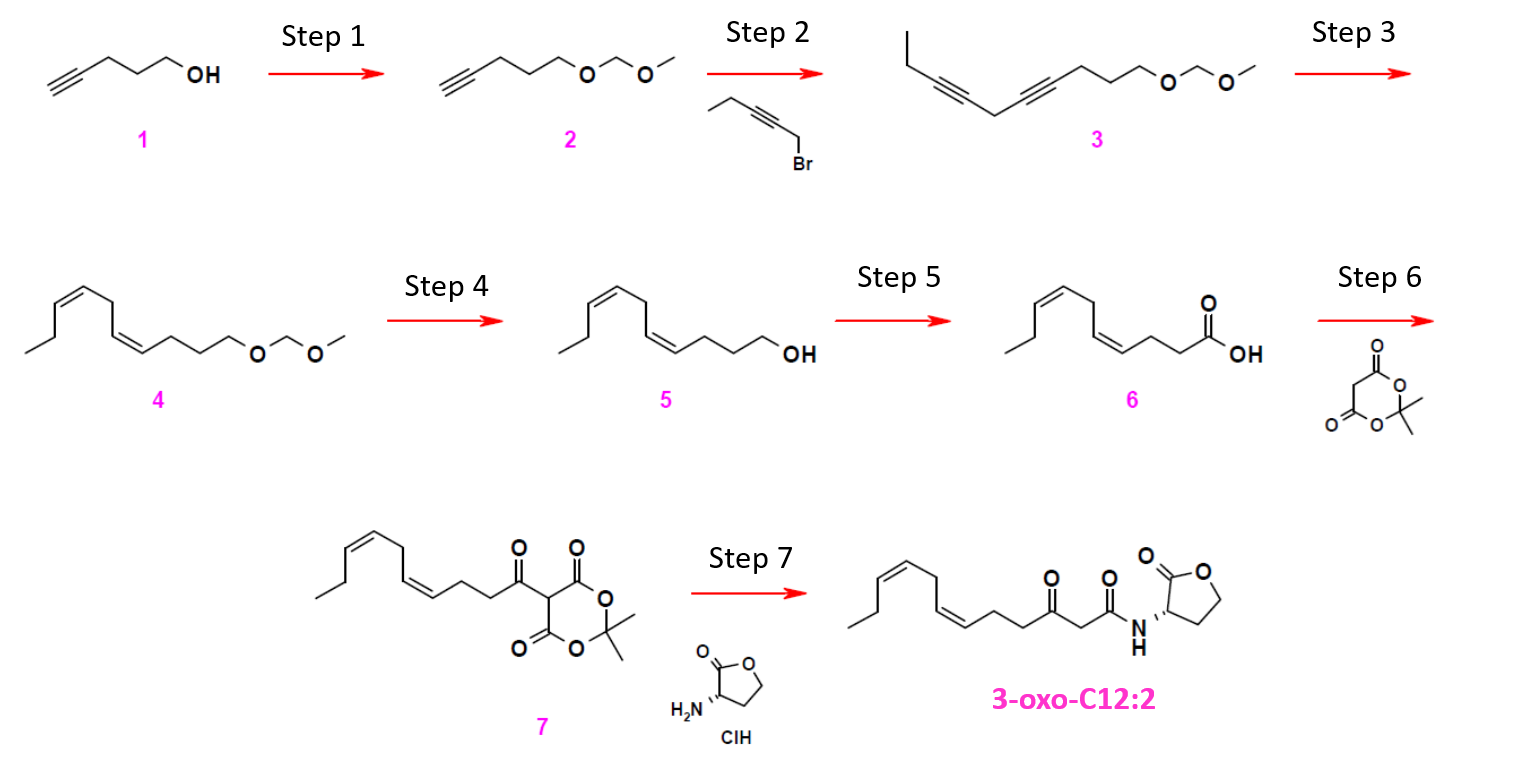

Supplement: S2 Fig — (TIF) [file pone.0202587.s004.tif]

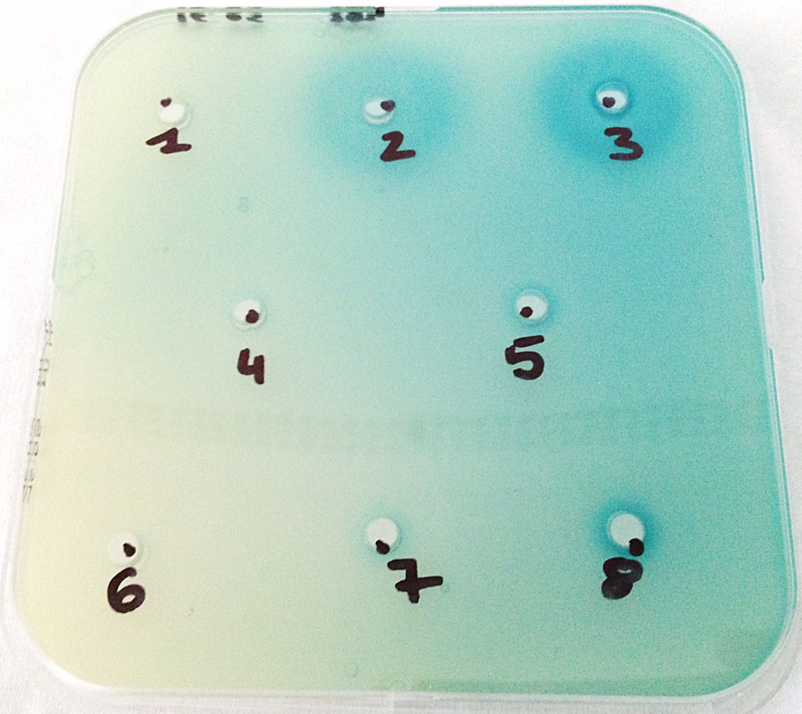

Supplement: S3 Fig — 1: control (water) 2: C7 5μM 3: 3-oxo-C12 5μM, HPLC fraction with AHL at m/z 294.2 with different concentrations : 4 : X1/10, 5 : X1/2, 6 : X1, 7 : X2.5, 8 : X5. (TIF) [file pone.0202587.s005.tif]

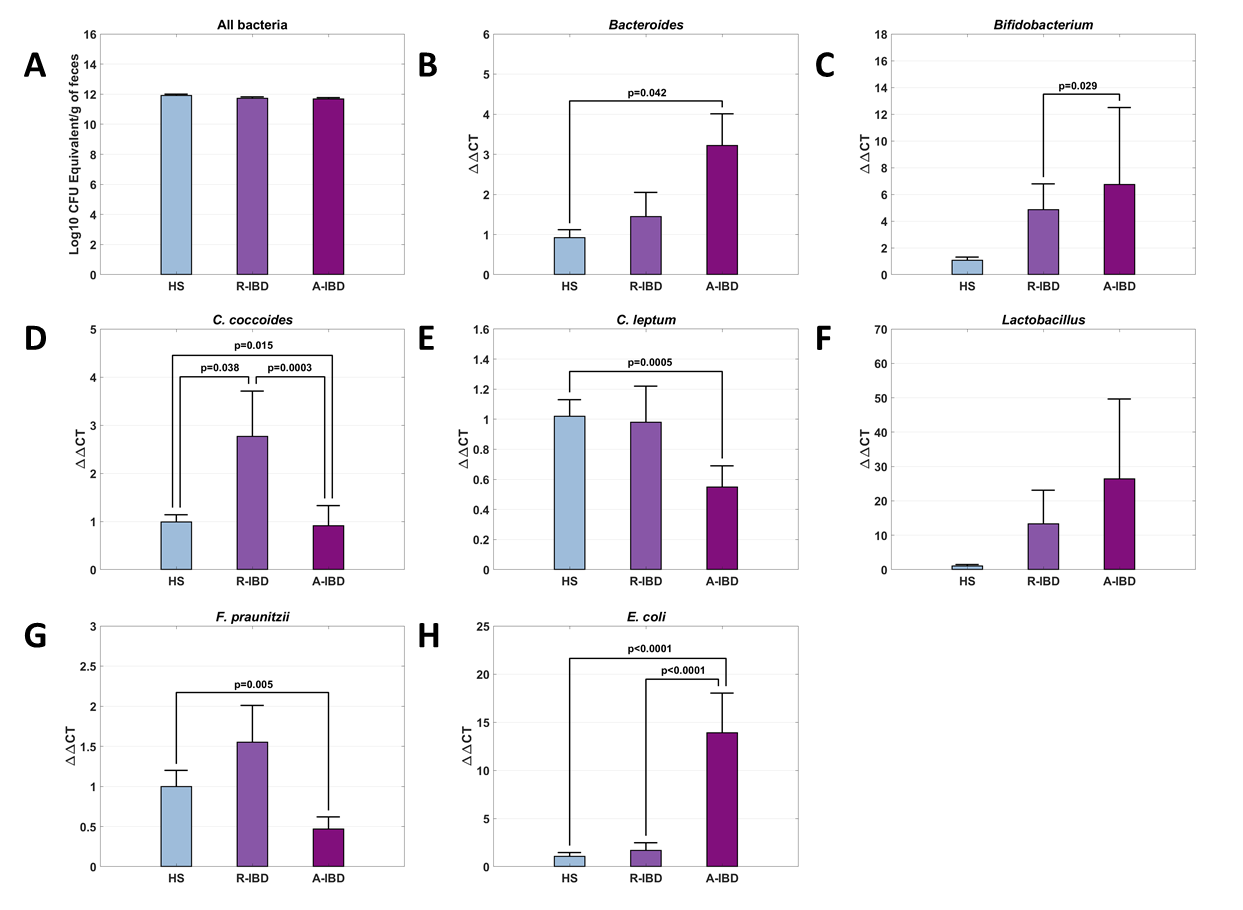

Supplement: S4 Fig — A: All bacteria in Log10 CFU Equivalent/g of feces (+/-SEM); B, C, D, E, F, G, H : ΔΔCT (+/-SEM) qPCR of dominant and subdominant bacterial groups and specific species in feces. Thin bars indicates significant comparisons. (TIF) [file pone.0202587.s006.tif]

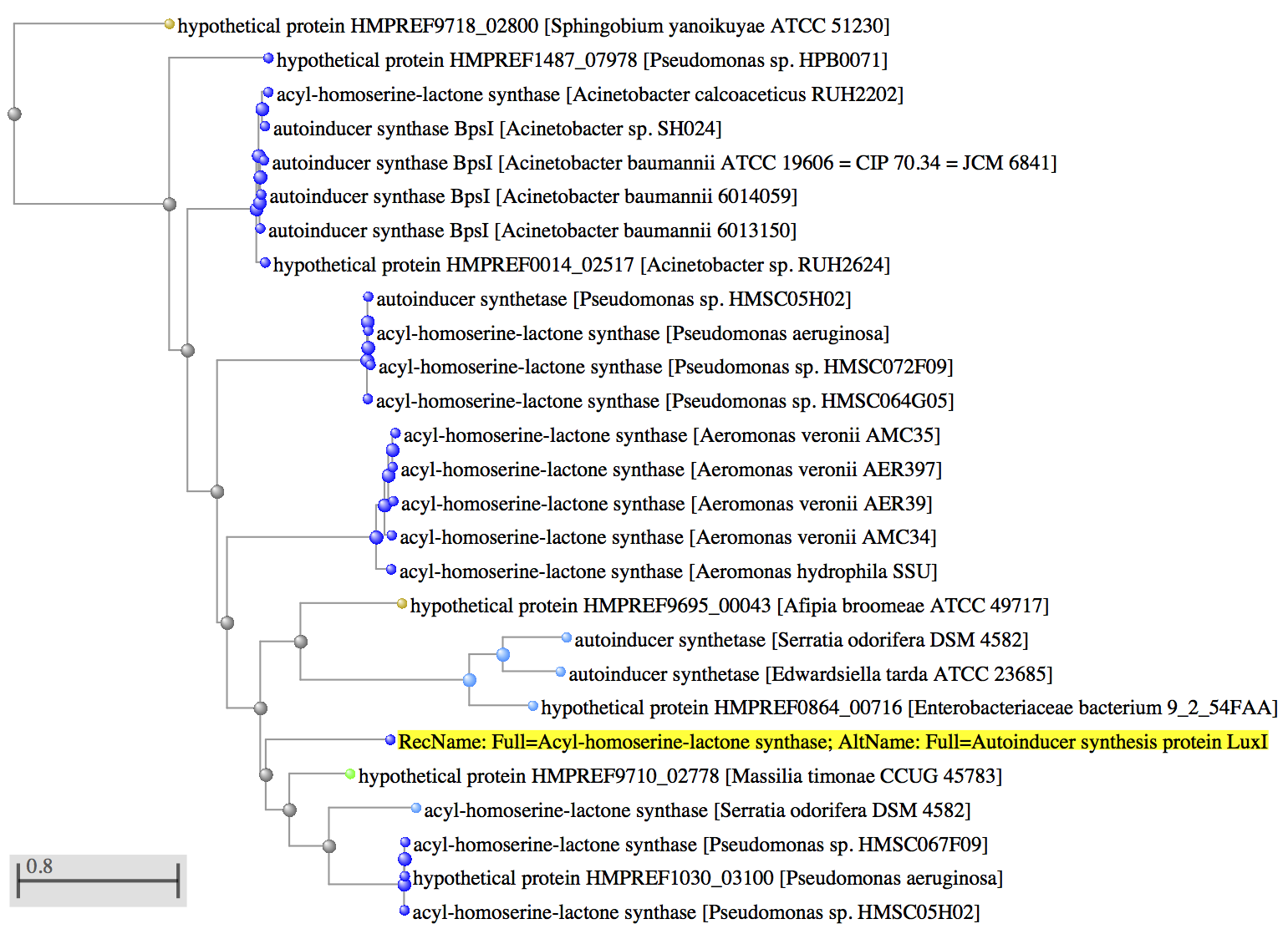

Supplement: S5 Fig — Phylogenetic tree was contructed with fast minimum evolution algorithm and maximum sequence difference of 0.85 with Grishin distance highlithed sequence corresponds to LuxI original sequence. Blast names color map : blue : g-proteobacteria; light blue: b-proteobacteria; pink : proteobacteria; red: bacteria; grey : a-proteobacteria; beige : d-proteobacteria; green : other sequences. (TIFF) [file pone.0202587.s007.tiff]

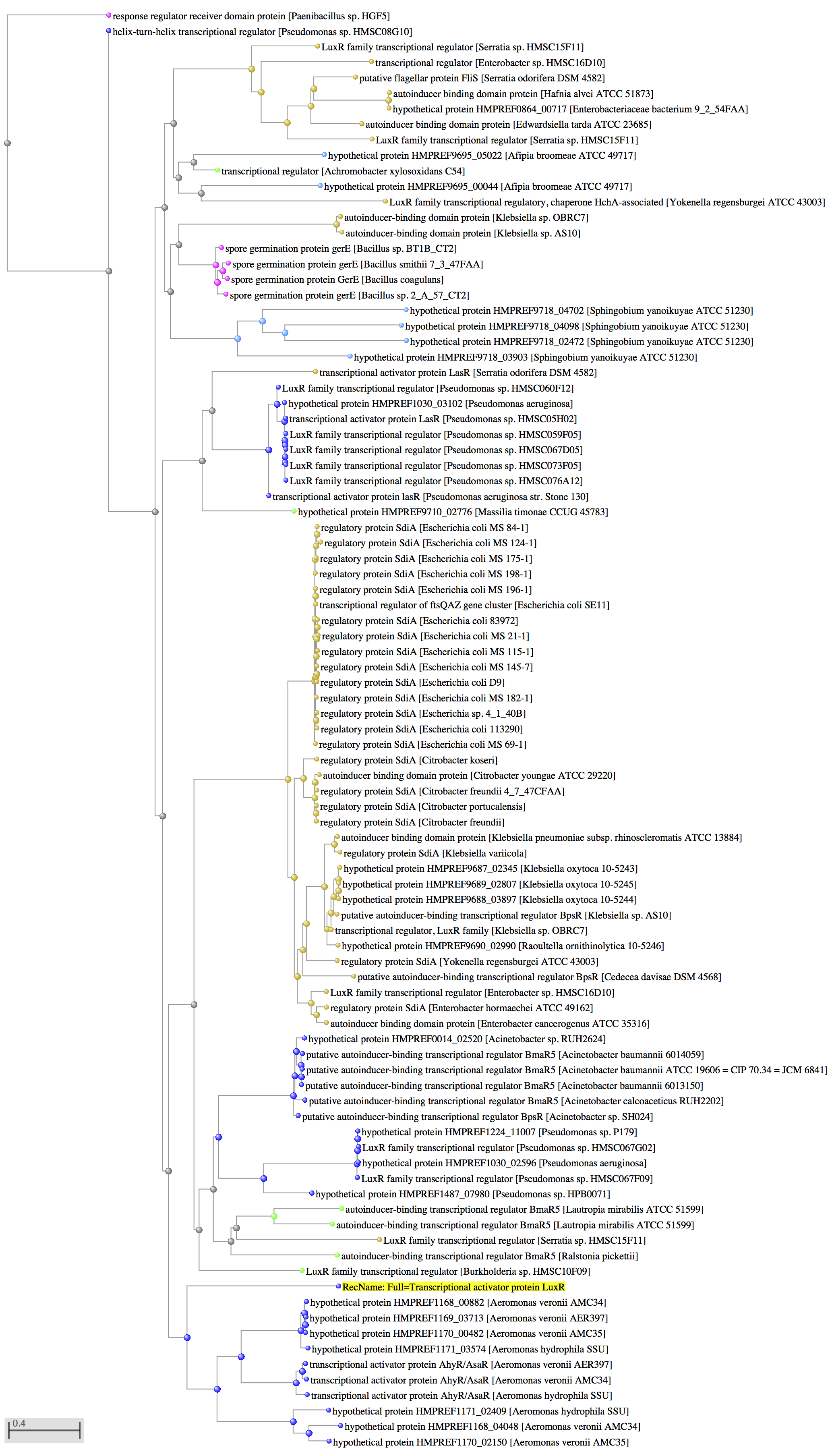

Supplement: S6 Fig — Phylogenetic tree was constructed with fast minimum evolution algorithm and maximum sequence difference of 0.85 with Grishin distance highlithed sequence corresponds to LuxR original sequence. Blast names color map : blue : g-proteobacteria; light blue: b-proteobacteria; pink : proteobacteria; red: bacteria; grey : a-proteobacteria; beige : d-proteobacteria; green : other sequences. (TIFF) [file pone.0202587.s008.tiff]
